# Sensory impairments associated with cognitive impairment among older adults in China: A community-based, 10-year prospective cohort study

**DOI:** 10.7189/jogh.14.04175

**Published:** 2024-10-04

**Authors:** Chao Yang, Ying Zhang, Huan Li, Xiao Ji, Huali Wang, Xiaozhen Lv

**Affiliations:** 1 Department of Psychiatry, Beijing Luhe Hospital, Capital Medical University, Beijing, China; 2 Beijing Key Laboratory of Mental Disorders, National Clinical Research Center for Mental Disorders & National Center for Mental Disorders, Beijing Anding Hospital, Capital Medical University, Beijing, China; 3 Advanced Innovation Center for Human Brain Protection, Capital Medical University, Beijing, China; 4 Beijing Dementia Key Lab, National Clinical Research Center for Mental Disorders, NHC Key Laboratory of Mental Health (Peking University), Peking University Institute of Mental Health (Sixth Hospital), Beijing, China

## Abstract

**Background:**

To address an existing gap in knowledge due to limited and inconclusive evidence, we aimed to investigate the association between sensory impairments and cognitive decline among older Chinese individuals.

**Methods:**

We retrieved data on 6862 adults aged ≥65 years that were collected through the Chinese Longitudinal Healthy Longevity Study (CLHLS), a nationwide, prospective, community-based elderly cohort study. Visual or hearing impairment in the CLHLS were identified through self-reported questionnaire. Sensory impairments were categorised as no sensory impairment, hearing impairment only, visual impairment only, and dual sensory impairment according to hearing and vision function. Cognitive impairment was defined as having a score <18 on the Chinese version of the Mini Mental State Examination. We used a Cox proportional hazard model to evaluate the relationship between sensory and cognitive impairments.

**Results:**

Among 6862 participants, 5.7% had dual sensory impairment, 7.4% had hearing impairment only, and had 17.2% visual impairment only. Compared with participants with no sensory impairment, those with hearing impairment only (adjusted hazard ratio (aHR) = 1.65; 95% confidence interval (CI) = 1.41, 1.92), visual impairment only (aHR = 1.25; 95% CI = 1.11, 1.41), and dual sensory impairment (aHR = 1.47; 95% CI = 1.25, 1.74) were significantly associated with higher risk of cognitive impairment in the fully adjusted model.

**Conclusions:**

Our results show that having hearing impairment only, visual impairment only, and dual sensory impairment was significantly associated with a higher risk of cognitive impairment among Chinese older adults aged ≥65 years. This suggest a need for the timely identification and management of sensory impairments for the elderly to reduce dementia risk.

Dementia is the main cause of disability among people >65 years of age worldwide, including in China [1,2], with epidemiological studies showing that the number of individuals suffering from dementia in China will reach 23.3 million by 2030 [3,4]. Likewise, an estimated 38 million Chinese elderly individuals >60 years of age suffer from cognitive impairment, which is considered a hallmark of dementia [5]. Unfortunately, no effective treatment exists for dementia, which makes identifying and understanding the potential risk factors associated with cognitive impairment, particularly modifiable risk factors, critical to delaying or reducing its onset.

Sensory impairments, comprising hearing, visual, and dual sensory impairments, are another serious concern among older individuals [6], as they may contribute to the risk of cognitive decline and pathological impairments, including dementia [7]. The currently predominant theory postulates that sensory impairments may not only increase a person’s cognitive load, but also lead to sequestration, reduced physical activity, and diminished social interaction. These factors may directly and indirectly increase the risk of dementia [8].

Previous studies have primarily focussed on the risk of poor cognition in relation to either visual or hearing impairment only in isolation, instead of dual sensory impairment [9–13], although they commonly co-occur among older individuals [14]. Dual sensory impairment, in turn, may pose a higher risk of cognitive impairment than any single sensory impairment due to the limited ability of individuals to compensate for the former through the functioning of an unimpaired sensory system. Despite this, previous research on the association between sensory impairments and cognitive decline has yielded contrasting results. Some studies have shown that older people with sensory impairments tend to experience more rapid cognitive decline or have a higher risk of dementia compared to those with normal sensory function [15–20]. These findings, however, contrast those of other research [21]. Different measures of sensory impairments, cognitive function, small samples, and variable lengths of follow-up may explain the conflicting results in previous studies. Moreover, most studies have focussed on high income individuals in developed countries. Compared with developed countries, individuals in China not only exhibit a notably higher prevalence of sensory impairments [22], but also differ significantly in some key risk factors associated with cognitive decline, such as economic status, race/ethnicity, lifestyle, and education. Unfortunately, there has been limited research on the association between hearing impairment only, visual impairment only, and dual sensory impairment and cognitive function concurrently in developing countries, especially in China. There are also few prospective studies on this topic among the Chinese elderly, as existing studies have used a cross-sectional design [22–25] and a single study focusing on younger participants [20]. While sensory impairments serve as risk factors for poor cognition, their relationship with cognitive impairment in older adults may be different from that observed in younger individuals, as the manifestation of these conditions with advancing age shows distinct and characteristic patterns [14,26]. Therefore, the roles of hearing impairment, visual impairment, or a combination thereof in predicting risk of cognitive impairment among older adults in China need so be further explored. To our knowledge, the association between sensory impairments and cognitive impairment has not been thoroughly evaluated among a national cohort of Chinese adults aged ≥65 years old, especially as few studies have examined age differences in sensory impairments and cognitive function.

To address this gap, we used data from the Chinese Longitudinal Healthy Longevity Study (CLHLS), a nation-wide, community-based prospective cohort of oldest adults in China with long-term follow up, to investigate the longitudinal association between sensory impairments and cognitive impairment. Furthermore, we sought to explore the impact of sensory impairments on cognitive impairment across different age subgroups in the older population.

## METHODS

### Data and study sample

We derived our data from the Chinese Longitudinal Healthy Longevity Study (CLHLS), a large-scale prospective community cohort study in China, launched in 1998 to investigate determinants of longevity in China. Since then, the follow-up surveys have been conducted every 2–3 years by trained interviewers with a structured questionnaire, who collected the data face-to-face from participants' homes. To ensure a representative sample, the CLHLS adopted a disproportionate, multi-stage, stratified cluster random sampling method, whereby the community-dwelling Chinese older adults were randomly selected from 866 counties and city districts from 22 of China’s 31 provinces. This makes the survey representative, as it covers approximately 85% of China’s older population [27,28]. Thorough evaluations encompassing the randomness of attrition, the credibility and validity of the measurement scale, as well as the precision of reported age have confirmed the validity of the CLHLS’ data [29,30].

**Figure 1:**
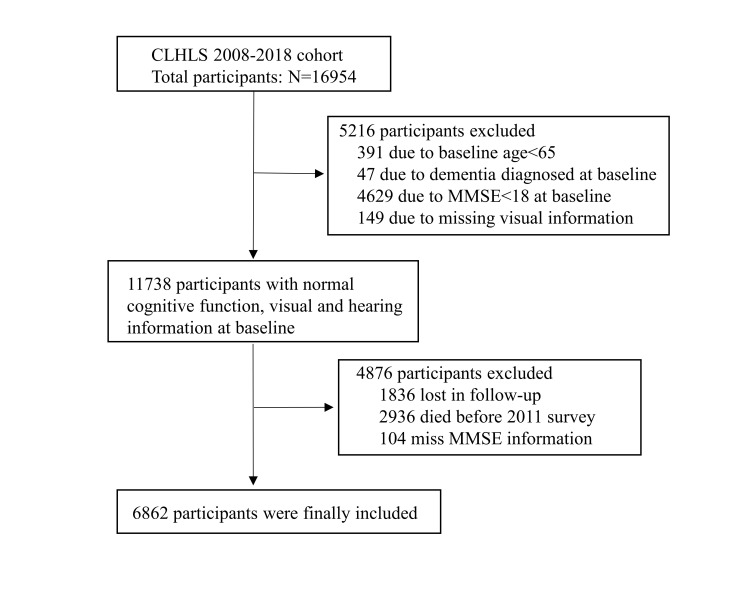
Flowchart of the study population from the CLHLS.

Here we used data from the 2008–18 cohorts spanning four survey waves (2008, 2011, 2014, and 2018). The 2008 wave of CLHLS included data on 16954 respondents. We initially retrieved data on 11738 participants aged ≥65 years who were free of dementia at baseline (Mini-Mental State Examination (MMSE) score ≥18). To be included in the final sample, the participants had to have completed visual and hearing assessments and the MMSE during any subsequent follow-up. Participants who were died or lost to follow-up or with missing value in MMSE were excluded before the first follow-up. Our final sample comprised 6862 participants (**Figure 1**).

### Assessment of sensory impairments

Visual and hearing functions were assessed based on participants’ responses to self-report questionnaires. Specifically, hearing function was assessed based on whether the participants could hear clearly what the interviewer said during the assessment, with the available responses being ‘Yes, without hearing aids’, ‘Yes, need a hearing aid’, ‘Partly, with hearing aid’, and ‘No’. The participants were categorised as having no hearing impairment only if they answered using the first response, and were categorised as having hearing impairment otherwise. With respect to visual function, the participants were showed a card with a flashlight shining on it and were asked ‘Without glasses, can you see if there is a break in the circle on the card and distinguish the direction of the notch in the circle?’ The possible responses included ‘Yes, can see and distinguish’, ‘Yes, can see but cannot distinguish’, ‘Cannot see clearly’, and ‘Blind’. The participants were identified as having visual impairment if they responding with the first option and were categorised as not having visual impairment otherwise.

Based on their reported sensory status at baseline, participants were grouped into four mutually exclusive sensory groups for analysis: no sensory impairment; visual impairment only; hearing impairment only; and dual sensory impairment (defined as participant having both hearing and visual impairment).

### Assessment of cognitive function

Cognitive function was measured by the Chinese version of the MMSE during each survey. The MMSE consists of 30 items referring to time orientation, location orientation, immediate memory, attention and calculation, delayed memory, language, and visual spatial ability; its validity and reliability have been determined previously [27,29,31]. The total score of MMSE ranges from 0 to 30, with higher scores indicating better cognitive function. Considering that the general education level of older people in China was illiteracy, MMSE score below 18 were defined as cognitive impairment [32–34]. This cut-off score demonstrates a specificity ranging from 80 to 100% and a sensitivity of 80 to 90% for diagnosing cognitive impairment among elderly Chinese adults [32,33]. Based on previous studies, the responses of ‘unable to answer’ was regarded as ‘wrong’ [32]. 

### Covariates

Based on existing literature [34,35], we adjusted our baseline model for some potential confounders, including sociodemographic characteristics, behavioural factors, and health conditions. The sociodemographic characteristics were age in years, sex, education (illiterate or literate), residence (urban or rural), living arrangement (alone or in nursing home or living with family), marital status (married or not married). Behavioural factors were current smoking (yes or no), current drinking (yes or no), current regular exercise (yes or no). Relevant health conditions were the body mass index (BMI) (underweight: <18.5 kg/m^2^, normal: 18.5–23.9kg/m^2^, overweight: 24.0–27.9 kg/m^2^; obese: ≥28.0 kg/m^2^ [36]), common chronic diseases (hypertension, diabetes, heart problems, cerebrovascular disease, and chronic lung diseases).

### Statistical analyses

We used the Kolmogorov-Smirnov test to check the normality of the distribution of our data. To compare baseline characteristics of the study participants across sensory groups, we used either analysis of variance or the Wilcoxon rank sum test for continuous variables (depending on the results of the normality test) and the χ^2^ test for categorical variables.

We then set up a Cox proportional hazards model to explore the longitudinal relationship between sensory impairments and cognitive impairment for 6862 participants. The endpoint event was defined as the first occurrence of cognitive impairment. The follow-up period started from baseline and ended on the date of cognitive impairment, death, or loss to follow-up, or the end of the study, whichever occurred first. Only education and BMI had missing values, comprising 0.2% participants for education and 0.5% for BMI. Considering the importance of education on cognitive impairment, we excluded those participants from the analytical sample. We otherwise used the mean BMI of our sample to handle the missing value of BMI. Aside from the initial unadjusted model (i.e., model 1), we further set up two Cox proportional hazards models that were adjusted for potential confounders, whereby model 2, was adjusted for sex, age, and education, and model 3 was further adjusted for residence, living arrangement, marital status, smoking, drinking, BMI, regular exercise, and chronic diseases (hypertension, diabetes, heart problems, cerebrovascular disease, chronic lung diseases). We presented the model results in the form of adjusted hazard ratios (aHRs) and 95% confidence intervals (CIs).

To analyse whether sensory impairments were associated with subsequent cognitive impairment, we examined longitudinal cognitive impairment (during the follow-up period) using linear mixed-effects models (LMM). We evaluated the association between baseline sensory impairments and MMSE score with ‘time from baseline (month)’ × ‘group (no sensory impairment; visual impairment only; hearing impairment only; dual sensory impairment.)’ interaction in the LMM. Here, covariates were inserted in the same order as in the Cox proportional hazards model.

Furthermore, we used another Cox proportional hazards model to explore the highest risks for developing cognitive impairment among individuals with visual impairment only, hearing impairment only, and dual sensory impairment. In this analysis, individuals with hearing impairment only and those with visual impairment only were taken as reference groups, according to which two sets of Cox proportional hazards models were estimated. We inserted the above-mentioned covariates in the same order as the main Cox proportional hazards model.

We performed further subgroup analyses to examine whether the association with sensory impairments varied by age (65–79 years or ≥80 years) and educational (illiterate or not). Effect modification was further identified through the inclusion of interaction terms between sensory impairments and the aforementioned variables in the multivariable model, respectively. We likewise performed multiple sensitivity analyses to assess the robustness of our results by first redefining cognitive impairment as an MMSE score <24 for analysis. To further reduce potential residual confounding with short follow-up, we conducted another sensitivity analysis by excluding data from first follow-up participants (2011 years).

A two-sided *P* < 0.05 denoted statistical significance. We analysed all data in SPSS, version 26.0 (IBM Corp., Armonk, New York, USA).

### Ethical approval

The Ethics Committee of Peking University (IRB00001052-13074) approved the CLHLS study protocol. Each participant or their caregiver gave written informed consent after receiving a complete explanation of the study at baseline and follow-up investigations.

## RESULTS

### Participant characteristics

The study sample consisted of 6862 participants (3513 women and 3349 men) (**Table 1**). The mean age at the baseline was 80.9 (standard deviation (SD) = 10.1) years old. Approximately 47.6% of participants were literate, while 48.0% were married. The mean baseline score of MMSE was 26.8 points (SD = 3.3). Moreover, 69.8% of participants had no sensory impairment at baseline, 7.4% had hearing impairment only, 17.2% had visual impairment only, and 5.7% had dual sensory impairment. In comparison with participants with no sensory impairment, a higher proportion of participants with visual impairment only, hearing impairment only, or dual sensory impairment tended to be older, female, illiterate, not married, living in the rural, and not do regular exercise.

**Table 1:** Characteristics of participants at baseline (n = 6862)*

	Total	No impairment	Hearing impairment only	Visual impairment only	Dual sensory impairment	*P*-value
**No. of participants**	6862 (100.0)	4788 (69.8)	506 (7.4)	1179 (17.2)	389 (5.7)	
**Age in years, x̄ (SD)**	80.9 (10.1)	78.7 (9.5)	88.3 (8.8)	83.6 (9.7)	89.8 (8.3)	<0.001
**Sex**						<0.001
Male	3349 (48.8)	2473 (51.6)	260 (51.4)	472 (40.0)	144 (37.0)	
Female	3513 (51.2)	2315 (48.4)	246 (48.6)	707 (60.0)	245 (63.0)	
**Education**						<0.001
Illiterate	3589 (52.4)	2245 (47.0)	319 (63.3)	736 (62.6)	289 (74.5)	
Literate	3257 (47.6)	2533 (53.0)	185 (36.7)	440 (37.4)	99 (25.5)	
**Marital status**						<0.001
Married	3297 (48.0)	2606 (54.4)	153 (30.2)	438 (37.2)	100 (25.7)	
Not married	3565 (52.0)	2182 (45.6)	353 (69.8)	741 (62.8)	289 (74.3)	
**Type of residence**						0.007
Urban	1220 (17.8)	899 (18.8)	75 (14.8)	192 (16.3)	54 (13.9)	
Rural	5642 (82.2)	3889 (81.2)	431 (85.2)	987 (83.7)	335 (86.1)	
**Living arrangement**						0.001
Alone or in nursing home	1238 (18.0)	806 (16.8)	95 (18.8)	252 (21.4)	85 (21.9)	
Living with family	5624 (82.0)	3982 (83.2)	411 (81.2)	927 (78.6)	304 (78.1)	
**Current smoking**						<0.001
Yes	1502 (21.9)	1161 (24.2)	107 (21.1)	196 (16.6)	38 (9.8)	
No	5360 (78.1)	3627 (75.8)	399 (78.9)	983 (83.4)	351 (90.2)	
**Current drinking**						<0.001
Yes	1419 (20.7)	1068 (22.3)	113 (22.3)	195 (16.5)	43 (11.1)	
No	5443 (79.3)	3720 (77.7)	393 (77.7)	984 (83.5)	346 (88.9)	
**Regular exercise**						<0.001
Yes	2470 (36.0)	1844 (38.5)	152 (30.0)	383 (32.5)	91 (23.4)	
No	4392 (64.0)	2944 (61.5)	354 (70.0)	796 (67.5)	298 (76.6)	
**BMI**						<0.001
Underweight (<18.5 kg/m^2^)	3890 (57.0)	2768 (58.0)	276 (55.6)	632 (53.7)	214 (55.4)	
Normal (18.5–24.0 kg/m^2^)	1759 (25.8)	1060 (22.2)	144 (29.0)	414 (35.2)	141 (36.5)	
Overweight (24.0–28.0 kg/m^2^)	953 (14.0)	770 (16.1)	63 (12.7)	96 (8.2)	24 (6.2)	
Obese (≥28.0 kg/m^2^)	226 (3.3)	172 (3.6)	13 (2.6)	34 (2.9)	7 (1.8)	
**Chronic diseases**						
Hypertension						0.544
*Yes*	1510 (22.0)	1073 (22.4)	102 (20.2)	248 (21.0)	87 (22.4)	
*No*	5352 (78.0)	3715 (77.6)	404 (79.8)	931 (79.0)	302 (77.6)	
Diabetes						0.650
*Yes*	206 (3.0)	151 (3.2)	12 (2.4)	31 (2.6)	12 (3.1)	
*No*	6656 (97.0)	4637 (96.8)	494 (97.6)	1148 (97.4)	377 (96.9)	
Heart problems						0.050
*Yes*	648 (9.4)	479 (10.0)	38 (7.5)	105 (8.9)	26 (6.7)	
*No*	6214 (90.6)	4309 (90.0)	468 (92.5)	1074 (91.1)	363 (93.3)	
Cerebrovascular disease						0.747
*Yes*	356 (5.2)	256 (5.3)	27 (5.3)	54 (4.6)	19 (4.9)	
*No*	6506 (94.8)	4532 (94.7)	479 (94.7)	1125 (95.4)	370 (95.1)	
Chronic lung diseases						0.598
*Yes*	659 (9.6)	461 (9.6)	51 (10.1)	117 (9.9)	30 (7.7)	
*No*	6203 (90.4)	4327 (90.4)	455 (89.9)	1062 (90.1)	359 (92.3)	
**MMSE score at baseline, x̄ (SD)**	26.8 (3.3)	27.4 (2.9)	24.7 (3.7)	26.0 (3.4)	24.0 (3.8)	<0.001
**Survival time in months, MD (IQR)**	69 (37–108)	70 (38–118)	38 (36–70)	66 (37–73)	38 (36–70)	<0.001
**Cognitive impairment in the end**	1712 (24.9)	925 (19.3)	224 (44.3)	378 (32.1)	185 (47.6)	<0.001

BMI – body mass index, IQR – interquartile range, MD – median, MMSE – Mini-Mental State Examinations, SD *–* standard deviation, x̄ – mean

*Presented as n (%) unless specified otherwise.

### Longitudinal association of sensory impairments with cognitive impairment

During the 10 years of follow-up (median follow-up of 5.7 years (interquartile range = 3.1–9.0)), 1712 (24.9%) participants were identified as cognitive impairment, with the incidence of cognitive impairment being the largest in those with dual sensory impairment (47.6%) compared to those with no sensory impairment (19.3%), visual impairment only (32.1%), hearing impairment only (44.3%).

**Figure 2:**
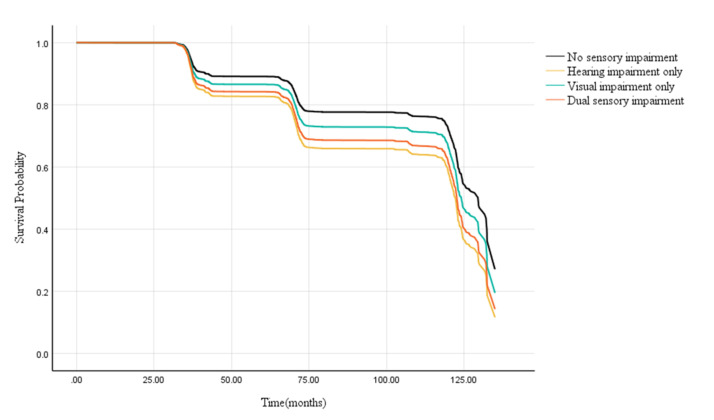
Kaplan-Meier curves for adjusted incident of cognitive impairment by status of sensory impairment.

According to the results of Cox proportional hazard analysis (**Table 2**), in comparison with no sensory impairment at baseline in model 3 after adjusting confounders, hearing impairment only (aHR = 1.65; 95% CI = 1.41, 1.92), visual impairment only (aHR = 1.25; 95% CI = 1.11, 1.41), and dual sensory impairment (aHR = 1.47; 95% CI = 1.25, 1.74) were significantly associated with a higher risk of incident cognitive impairment. Additionally, we observed a significantly higher risk of cognitive impairment among individuals with hearing impairment only (*P* < 0.001) and those with dual sensory impairment (*P* = 0.007) compared to those with visual impairment only. However, compared to hearing impairment only, dual sensory impairment did not show a statistically significant difference in the risk of cognitive impairment (*P* = 0.29) (Table S1 in the **Online Supplementary Document**). The Kaplan-Meier curves for adjusted incident of cognitive impairment (**Figure 2**) showed differences in risk based on the presence of sensory impairments.

**Table 2:** Association between baseline sensory impairments and the incidence of cognitive impairment*

	HR (95% CI)		
**Status of sensory impairment**	**Model 1**	**Model 2**	**Model 3**
No sensory impairment	ref	ref	ref
Hearing impairment only	3.68 (3.18, 4.27)	1.70 (1.46, 1.97)	1.65 (1.41, 1.92)
Visual impairment only	1.96 (1.74, 2.22)	1.28 (1.13, 1.44)	1.25 (1.11, 1.41)
Dual sensory impairment	4.05 (3.46, 4.75)	1.54 (1.31 1.82)	1.47 (1.25, 1.74)

CI – confidence interval, HR – hazard ratio, ref – reference

*Model 1 – unadjusted model. Model 2 – adjusted for age, sex, and education. Model 3 – adjusted for age, sex, education, type of residence, living arrangement, marital status, smoking, drinking, body mass index, regular exercise, and chronic diseases (hypertension, diabetes, heart problems, cerebrovascular disease, chronic lung diseases).

### Association between sensory impairments and MMSE scores during follow-up

In the LMM analysis with MMSE scores as outcome variable, the overall MMSE scores decreased after follow-up (correlation coefficient (*β*) = −0.035; 95% CI = −0.038, −0.032, *P* < 0.001). There was a statistically significant time interaction with hearing impairment only (*β* = −0.048; 95% CI = −0.058, −0.038, *P* < 0.001), visual impairment only (*β* = −0.015; 95% CI = −0.021, −0.009, *P* < 0.001), and dual sensory impairment (*β* = −0.037; 95% CI = −0.048, −0.025, *P* < 0.001), even after adjusting all confounding factors. These results indicated a significant faster decline in the MMSE scores among individuals with sensory impairments as compared to those without it during the follow-up period (**Table 3**).

**Table 3:** Longitudinal cognitive decline for participants with sensory impairments compared with participants without sensory impairments*

Predictors	β (95% CI)	*P*-value
Model 1		
*Hearing impairment × time*	−0.049 (−0.060, −0.029)	<0.001
*Visual impairment × time*	−0.015 (−0.021, −0.009)	<0.001
*Dual sensory impairment × time*	−0.04 (−0.052, −0.028)	<0.001
Model 2		
*Hearing impairment × time*	−0.048 (−0.058, −0.038)	<0.001
*Visual impairment × time*	−0.015 (−0.021, −0.009)	<0.001
*Dual sensory impairment × time*	−0.036 (−0.048, −0.025)	<0.001
Model 3		
*Hearing impairment × time*	−0.048 (−0.058, −0.038)	<0.001
*Visual impairment × time*	−0.015 (−0.021, −0.009)	<0.001
*Dual sensory impairment × time*	−0.037 (−0.048, −0.025)	<0.001

*We used ‘no sensory impairment’ as the reference. Time was expressed in months. Model 1 – unadjusted model. Model 2 – adjusted for age, sex, and education. Model 3 – adjusted for age, sex, education, type of residence, living arrangement, marital status, smoking, drinking, body mass index, regular exercise, and chronic diseases (hypertension, diabetes, heart problems, cerebrovascular disease, chronic lung diseases).

### Subgroup analysis

**Figure 3:**
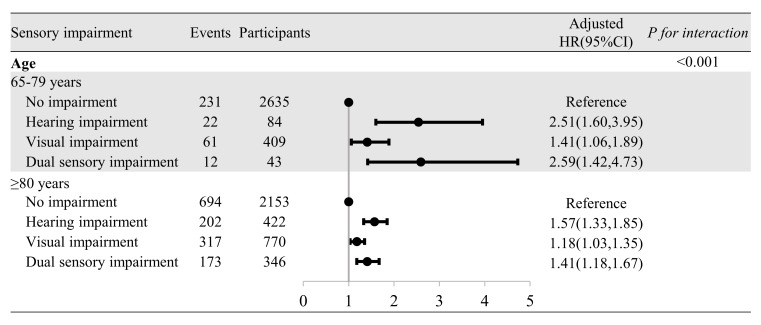
Subgroup analysis for the association of baseline sensory impairments with cognitive impairment. Subgroup analysis by age was adjusted for the following covariates sex, education, type of residence, living arrangement, marital status, smoking, drinking, BMI, regular exercise, and chronic diseases. CI – confidence interval, HR – hazard ratio.

We observed similar results for baseline sensory impairments across age-based subgroups (**Figure 3**). The risk of cognitive impairment remained notably higher for individuals with sensory impairments than those with no sensory impairment after the stratified analysis by age (65–79 years and ≥80 years old). Among individuals aged 65–79 years old, the risk was significantly higher for those with hearing impairment only (aHR = 2.51; 95% CI = 1.60, 3.95), visual impairment only (aHR = 1.41; 95% CI = 1.06, 1.89), and dual sensory impairment (aHR = 2.59; 95% CI = 1.42, 4.73) compared to those with no sensory impairment. Similarly, the increased risk persisted with hearing impairment only (aHR = 1.57; 95% CI = 1.33, 1.85), visual impairment only (aHR = 1.18; 95% CI = 1.03, 1.35), and dual sensory impairment (aHR = 1.41; 95% CI = 1.18, 1.67) for those aged ≥80 years old. The associations of sensory impairments and cognitive impairment were non-significantly different across subgroups of education (*P*-value for interaction = 0.7).

### Sensitivity analysis

The findings in our sensitivity analysis remained consistent with those of the primary analyses even after participants lost to follow-up during the year 2011 were excluded and a MMSE score of 24 was employed as the threshold for defining cognitive impairment (Table S2 in the **Online Supplementary Document**).

## DISCUSSION

In this community-focussed, population-based prospective cohort study, we observed that hearing impairment only, visual impairment only and dual sensory impairment were associated with a higher likelihood of incident cognitive impairment over 10 years of follow-up among older individuals aged ≥65 years in China. These findings remained robust in our sensitivity analyses, while the subgroup analysis showed that cognitive decline was associated with different types of sensory impairments in groups stratified by age. Our findings extend the understanding of associations by examining not only hearing or visual impairment in isolation, but also dual sensory impairment through longitudinal data among China population, making it (to our knowledge) one of the first studies of this kind.

Our finding showed that participants with hearing impairment only were at an increased risk of cognitive decline compared with those with no sensory impairment, which is consistent with the results of previous research [13,17,19]. Notably, a 24-year longitudinal cohort study showed hearing impairment was associated with poorer performance and accelerated decline on measures of global cognitive function in a large community-dwelling cohort of older adults [13]. Similarly, hearing impairment was associated with faster rate of cognitive decline among 295 aged ≥73 years in the Health and Retirement Study (HRS) and its supplement, the Aging, Demographics, and Memory Study (ADAMS) [17]. However, several prior studies failed to find significant associations between hearing impairment only and cognitive performance [10,16,21]. This difference could stem from certain studies using smaller sample size [10], different definition criteria of cognitive function [21], and characteristics of participants [16].

Moreover, several studies reported a significant association between visual impairment only and cognitive impairment [9,11,18], although others found contradictory evidence [16,17,21]. The Korean Longitudinal Study on Cognitive Aging and Dementia [16] reported no significant associations between visual impairment only and cognitive decline among 6520 elderly individuals during the six-year follow-up. In another study from HRS and ADAMS, objectively measured visual impairment was not significantly associated with the rate of cognitive decline compared to no impairment [17]. Those studies differed from our study in terms of characteristics of the study populations, sample sizes, and measurements of sensory function.

Furthermore, we found that older adults with dual sensory impairment had a higher risk of cognitive decline than those with no sensory impairment. These findings are consistent with some previous studies [15,16,18] and opposed to others [21]. For example, Hong et al. [21] found htat dual sensory impairment was not significantly related to cognitive decline, yet they did not adjust for potential confounding actors such as education, BMI, smoking, alcohol consumption, daily exercise, and others, which could have led to biased results. Variations in the participants’ economic conditions, along with differences in the duration of follow-up, could also account for these discrepancies. It is challenging to pinpoint a key factor responsible for these inconsistent findings due to the limited number of studies available. Future research should consider employing multiple assessments of cognitive and sensory functions to better elucidate the longitudinal relationship between sensory impairments and cognitive decline.

The mechanisms underlying the association between sensory impairments and cognitive decline are largely unknown. Several hypotheses, including the sensory deprivation hypothesis, the high cognitive load caused by sensory impairments hypothesis, the information degradation hypothesis, and the common cause hypothesis propose various mechanisms that might explain the link between sensory impairments and cognitive decline [37]. The observed associations between sensory impairments and cognitive impairment may arise from structural and functional changes in the brain, given that decreases in grey matter density and reductions in temporal lobe volume were observed in patients with sensory impairments [38,39]. This change and decrease in cortical volume can be accompanied by cognitive aging or neurodegenerative diseases [40,41]. Unfortunately, the precise mechanisms underlying the association between sensory impairments and cognitive decline have not been determined. Future research should include more frequent longitudinal follow-up studies to determine whether there is a causal relationship between sensory impairments and cognitive decline, and further elucidate its potential pathological mechanisms.

In terms of study strengths, this is, to our knowledge, the first longitudinal study to investigate the relationship between sensory impairments and cognitive impairment in a national sample of community-dwelling older adults aged ≥65 years in China, with a mean age of 81 years which places them in a high-risk group for dementia. As China is a developing country, our results provide information for other, similar contexts, thereby addressing an existing research gap and highlighting that preserving sensory function could be a crucial intervention strategy for mitigating cognitive decline. Lastly, the conduct of the subgroup and sensitivity analyses further confirmed the robustness of our findings. 

Our study also has several limitations. First, sensory impairments were evaluated through self-reporting, rather than objective clinical assessments, which may have underestimated the prevalence of sensory impairments and, consequently, the association between sensory impairment and cognitive impairment. However, self-reported assessments of sensory function have been widely used in epidemiological studies [22–25], as prior studies indicated their high reliability and validity [42,43]. Second, air pollution and traumatic brain injury could be important risk factors for cognitive impairment in older people [1]. Yet, due to constraints in data availability, we could not include these covariates in our analysis. Further studies could address this gap by including confounders such as air pollution, various nutritional supplements, traumatic brain injury. and medication types. Third, although we have established that sensory impairments increase the risk of cognitive impairment, it remains uncertain whether preventing and treating sensory disorders could prevent the onset of dementia – a gap which would require further interventional studies. Finally, the apolipoprotein E genotype is a possible non-modifiable risk factor for cognitive impairment, which we could not include in our study due to constraints in data availability. However, it is worth noting that prior research has not identified any interaction between said genotype and sensory impairments [16].

## CONCLUSIONS

Our findings suggest that hearing impairment only, visual impairment only, and dual sensory impairments are linked to an increased likelihood of cognitive decline among elderly Chinese individuals. Our findings contribute to accumulating evidence that sensory impairments could serve as potential biomarker and modifiable risk factors for cognitive impairment, undescoring the significance of preserving auditory and visual health in China's aging population. More longitudinal studies with more frequent follow-up periods are needed in the future to explore the relationship between sensory impairments and cognitive impairment, especially randomised clinical trials which could examine whether interventions aimed at preventing or mitigating sensory impairments could help prevent future cognitive impairment.

## Additional material

Online Supplementary Document.
