# Supplementary material for: Sensory impairments associated with cognitive impairment among older adults in China: A community-based, 10-year prospective cohort study
Source: J Glob Health. 2024 Oct 4;14:04175. doi: 10.7189/jogh.14.04175 (PMC11450429; doi:10.7189/jogh.14.04175)
Supplement: Online Supplementary Document. [file jogh-14-04175-s001.pdf]

ONLINE SUPPLEMENTARY DOCUMENT

Title: Sensory impairments associated with cognitive impairment among older adults in China: A community-based, 10-year prospective cohort study

Authors: Chao Yang, Ying Zhang, Huan Li, Xiao Ji, Huali Wang, Xiaozhen Lv
*Corresponding author: Prof. Xiaozhen Lv, Dementia Care & Research Center, Peking University Institute of Mental Health, No.51 Huayuanbei Road, Beijing, 100191, China. E-mail: lvxiaozhen@bjmu.edu.cn.

Contents

Table S1. The association between sensory impairment and cognitive impairment under different reference variables. Page 2

Table S2: Sensitivity analysis for sensory impairment and cognitive impairment. Page 3

Table S1. The association between sensory impairment and cognitive impairment under different reference variables.

Main independent variable*	Adjusted HR (95% CI)	*P*
Model A		
Visual impairment	1 (reference)	-
Hearing impairment	1.44 (1.21-1.72)	<0.001
Dual sensory impairment	1.29 (1.07-1.56)	0.007
Model B		
Hearing impairment	1 (reference)	-
Visual impairment	0.69 (0.58-0.83)	<0.001
Dual sensory impairment	0.90 (0.73-1.10)	0.29

*Adjusted for age, sex, education, type of residence, living arrangement, marital status, smoking, drinking, body mass index, regular exercise, and chronic diseases (hypertension, diabetes, heart problems, cerebrovascular disease, chronic lung diseases);

HR= hazard ratios; CI= confidence intervals.

Table S2: Sensitivity analysis for sensory impairment and cognitive impairment.

Cognitive impairment	Events	Participants	Adjusted HR (95% CI) *
**Sensitivity analysis 1: Defined as cognitive impairment less than MMSE 24 (N=5297)**			
No impairment	1526	4033	1 (reference)
Hearing impairment	172	268	1.56(1.32,1.86)
Visual impairment	432	816	1.18(1.05,1.33)
Dual sensory impairment	129	180	1.36(1.11,1.66)
**Sensitivity analysis 1: Excluding data from participants in the first follow-up (2011 years) (N=5965)**			
No impairment	527	4261	1 (reference)
Hearing impairment	98	408	1.61(1.28,2.03)
Visual impairment	194	985	1.22(1.03,1.45)
Dual sensory impairment	78	311	1.51(1.17,1.95)

* Adjusted for age, sex, education, type of residence, living arrangement, marital status, smoking, drinking, body mass index, regular exercise, and chronic diseases (hypertension, diabetes, heart problems, cerebrovascular disease, chronic lung diseases).

HR= hazard ratios; CI= confidence intervals.
